# The Effects of Lycopene on the Growth Performance, Antioxidant Capacity, and Liver Health of Hybrid Groupers (♀ *Epinephelus fuscoguttatus* × ♂ *E. lanceolatus*) Under the Influence of Aflatoxin B1

**DOI:** 10.3390/ani16152330

**Published:** 2026-07-30

**Authors:** Yuxuan Han, Yilin Yao, Yansheng Liu, Yubin Liu, Xiaohui Dong

**Affiliations:** 1 Laboratory of Aquatic Animal Nutrition and Feed, College of Fisheries, Guangdong Ocean University, Zhanjiang 524088, China; 15945497497@163.com (Y.H.);; 2 Guangdong Engineering Technology Research Center of Accurate Nutrition and High-Efficiency Feeding of Aquatic Animals, Zhanjiang 524088, China

**Keywords:** lycopene, aflatoxin B1, growth performance, antioxidant capacity, liver health

## Abstract

The continued rise in fishmeal prices and the scarcity of fishery resources have led to an increase in the use of plant-based protein sources in compound feed, making mold contamination in compound feed a frequent occurrence. Aflatoxin B1 (AFB1) contamination in aquaculture feed can seriously harm fish growth and liver health; it is extremely toxic, posing an even greater risk to carnivorous fish such as grouper. Lycopene, a natural carotenoid, has antioxidant and anti-inflammatory physiological functions and may act as a functional feed additive to mitigate damage induced by mycotoxins. This study utilized hybrid grouper (*Epinephelus fuscoguttatus* ♀ × *E. lanceolatus* ♂) as the experimental model. An 8-week feeding trial was performed to systematically assess the impact of lycopene supplementation at levels of 0, 200, 400, 600, and 800 mg/kg to a basal diet containing 800 μg/kg AFB1 on the fish’s growth performance, antioxidant capacity, liver health, inflammation-related gene expression, and hepatic transcriptome. The results showed that adding 200 mg/kg lycopene to the feed significantly increased the FWB and SGR (*p* < 0.05) of hybrid grouper. An appropriate amount of lycopene could enhance the antioxidant capacity of serum and liver, significantly increase the activities of catalase (CAT) and superoxide dismutase (SOD), and significantly decrease serum alanine aminotransferase (ALT) activity while increasing albumin (ALB) levels (*p* < 0.05). An amount of 200 mg/kg lycopene significantly alleviated AFB1-induced hepatocyte vacuolation, steatosis, and inflammatory cell infiltration, while the protective effect of higher doses was weakened. The gene expression results showed that 200 mg/kg lycopene significantly upregulated the expression of antioxidant-related genes such as *cat*, *sod*, and *nrf-2*, and increased the expression level of the anti-inflammatory factor *il10* (*p* < 0.05). The transcriptome analysis identified a total of 621 differentially expressed genes, which were mainly enriched in metabolic pathways, fatty acid metabolism, PPAR signaling pathway, redox process, ferroptosis, NF-κB signaling pathway, and Toll-like receptor signaling pathway. In summary, the addition of 200 mg/kg lycopene to the feed can effectively alleviate the growth inhibition and liver damage caused by AFB1 in hybrid grouper. Its mechanism of action may be related to enhancing antioxidant defenses, improving liver tissue structure, regulating lipid metabolism, and maintaining immune homeostasis. The research results indicate that lycopene can be used as a natural functional feed additive to alleviate AFB1 toxicity in hybrid grouper aquaculture.

## Simple Summary

Aflatoxin B1 (AFB1) is the primary form of aflatoxin and is characterized by its high toxicity and carcinogenicity, posing a significant threat to the liver health of aquatic animals. The hybrid grouper (*Epinephelus fuscoguttatus* ♀ × *E. lanceolatus* ♂) is a commercially valuable fish species that is widely cultivated throughout the coastal regions of Southeast Asia. In this study, we investigated the capacity of lycopene—a natural, non-toxic antioxidant to mitigate the growth retardation and hepatotoxicity induced by AFB1 in hybrid grouper. In the experiment, it was found that the addition of 200 mg/kg of lycopene to the AFB1 feed effectively enhanced the growth performance, antioxidant capacity, and liver health of hybrid grouper. However, further increasing the lycopene concentration exacerbated liver damage. Experiments have demonstrated that lycopene serves as a natural feed additive capable of counteracting the negative impacts of aflatoxin B1, thereby improving the growth performance of fish and enhancing their hepatic health.

## 1. Introduction

Born from the union of female brown-marbled grouper (♀ *Epinephelus fuscoguttatus*) and male giant grouper (♂ *E. lanceolatus*), the hybrid grouper stands as a remarkable hybrid in aquaculture, it is characterized by its strong disease resistance, rapid growth rate, high-quality meat, significant economic benefits, and substantial growth potential [1]. It is now one of the primary high-value species for intensive aquaculture, including both factory farming and cage farming, in Southern China [2]. However, in an intensive aquaculture environment, grouper are more sensitive to toxins from feed sources, leading to frequent liver health problems [3].

As the global aquaculture industry intensifies, the increasing scarcity and high cost of fishmeal have emerged as critical bottlenecks to its sustainable development. Consequently, the partial replacement of fishmeal with plant-based protein sources has become a primary approach in the feed industry [4]. Mycotoxins are widespread in over 90% of the 426 known global plant feed ingredients, particularly in plant protein sources like soybeans [5]. Especially in southern regions of China, the prolonged high temperature and high humidity create conditions where plant feed raw materials are more susceptible to mold spoilage, thereby significantly elevating the risk of fungal proliferation and mycotoxin generation [6].

Aflatoxin is a secondary metabolite produced by filamentous fungi, notably *Aspergillus flavus* and *Aspergillus parasiticus*, and is considered the most toxic class of mycotoxins [7]. AFB1 has garnered considerable attention due to its extreme toxicity and potent carcinogenicity. Its crystalline form exhibits high thermal stability, rendering it resistant to removal by standard feed processing methods [8]. AFB1 is a potent carcinogen, as well as a teratogen and mutagen. It enters the food chain directly by contaminating crops, or indirectly through animal products derived from livestock that have ingested contaminated feed, thereby presenting a dual hazard to the health of both humans and animals [9]. AFB1 enters liver cells, it is metabolized and activated by cytochrome P450 enzymes into the highly reactive AFB1-8,9-epoxide, which can bind to DNA, RNA, and proteins and interfere with normal cellular functions. The metabolism of AFB1 also promotes the excessive production of reactive oxygen species and depletes glutathione, thereby damaging the enzymatic antioxidant defense system, including superoxide dismutase, catalase, and glutathione peroxidase. Oxidative damage and mitochondrial damage may further activate redox-sensitive inflammatory pathways, including Toll-like receptor and NF-κB-related signaling, leading to the production of pro-inflammatory cytokines [10,11,12]. Compared to their omnivorous counterparts, carnivorous fish like *Lateolabrax maculatus* exhibit greater sensitivity to AFB1 and diminished tolerance [13]. The biological impacts of AFB1 on aquatic animals are primarily determined by the AFB1 concentration in their diet, in addition to factors such as the animal’s species, developmental stage, nutritional condition, and surrounding environment [14]. In previous studies on hybrid grouper, hybrid groupers fed diets containing 0, 7, 30, 111, 445, or 2230 μg/kg AFB1 for eight weeks showed progressively reduced growth performance and feed utilization and increased serum indicators of liver injury as the dietary AFB1 concentration increased. High dietary AFB1 concentrations also induced hepatic reactive oxygen species accumulation and inflammatory responses, weakened antioxidant defenses, and disrupted hepatic protein and lipid metabolism [3]. Further investigation showed that AFB1 toxicity was not restricted to the liver but was also associated with impaired intestinal health and muscle quality in hybrid grouper. Histological and multi-omics analyses subsequently demonstrated that dietary AFB1 concentrations of 445 μg/kg or higher caused growth inhibition, liver injury, hepatic AFB1 accumulation, and reductions in muscle polyunsaturated fatty acids. The associated metabolic alterations mainly involved retinol metabolism, cytochrome P450-mediated xenobiotic metabolism, and the biosynthesis of unsaturated fatty acids [15]. Collectively, these studies indicate that dietary AFB1 compromises grouper health through interconnected oxidative, inflammatory, intestinal, and metabolic disturbances. Nevertheless, nutritional strategies for mitigating AFB1 toxicity in grouper, particularly those involving natural antioxidant feed additives, remain insufficiently investigated. The primary toxicological target organ of AFB1 is the liver, and its molecular mechanisms underlying hepatocyte injury and hepatocarcinogenesis have been extensively investigated. Furthermore, recent studies have shown that AFB1-induced hepatotoxicity also involves multiple pathophysiological pathways such as mitochondrial dysfunction, oxidative stress, and inflammatory responses; these mechanisms are intertwined and jointly aggravate liver injury [16].

Lycopene is a naturally occurring carotenoid that is abundant in tomatoes, watermelons, guavas, and other red-colored fruits and vegetables [17]. Owing to its highly conjugated structure, lycopene possesses strong free-radical-scavenging capacity and has been widely investigated for its antioxidant, anti-inflammatory, immunomodulatory, and metabolism-regulating properties [18]. Mechanistically, lycopene may protect tissues by enhancing endogenous antioxidant defenses, regulating redox-sensitive signaling pathways, and suppressing excessive inflammatory responses. The protective effects of lycopene against toxicant-induced injury have been demonstrated in several animal models. In mammals, lycopene supplementation has been reported to alleviate AFB1-induced hepatic, renal, and reproductive toxicity by reducing oxidative stress, inflammation, and mitochondrial dysfunction [19]. Evidence from aquatic animals also suggests that lycopene can improve antioxidant status and protect against environmentally or nutritionally induced damage. For example, dietary lycopene alleviated sulfamethoxazole-induced hepatotoxicity in *Ctenopharyngodon idella* by suppressing oxidative stress, inflammation, and apoptosis [20]. Lycopene-containing diets have also been associated with improved antioxidant capacity and physiological condition in *Litopenaeus vannamei* [21], and tomato-derived carotenoids have been shown to reduce cadmium-induced biochemical and hematological disturbances in *Nile tilapia* [22]. These findings indicate that lycopene has potential as a functional feed additive for protecting aquatic animals against oxidative and hepatic injury.

Nevertheless, information regarding the use of lycopene to alleviate mycotoxin-induced toxicity in marine fish remains limited, and its protective effects against AFB1-induced liver injury in hybrid grouper have not been clearly established. Prior studies have confirmed that AFB1 induces significant physiological damage in hybrid groupers [3]. Building upon this foundation, the present study investigates the effects of different levels of lycopene under controlled conditions of equivalent nitrogen, lipid, and AFB1 concentrations. By employing a range of methodologies—including the assessment of growth performance, analysis of serum biochemical and immune parameters, histopathological examination of liver tissue, evaluation of antioxidant capacity, quantification of key inflammatory gene expression, and liver transcriptome sequencing—this research aims to elucidate the efficacy of lycopene in mitigating AFB1-induced damage and to determine its optimal dosage. The findings are intended to provide a robust theoretical framework for the development of functional feed additives designed to counteract mycotoxin contamination.

## 2. Materials and Methods

### 2.1. Ethics Statement

All fish used in this experiment were handled in accordance with the ethical guidelines established by the Animal Ethics Committee of Guangdong Ocean University, following procedures that were both scientific and standardized (Approval number: GDOU-IACUC-2025-A0728).

### 2.2. Preparation of Experimental Diets

Table 1 presents the experimental diet formulation and its nutrient composition. In this experiment, five experimental diets with equal nitrogen, equal fat, and equal AFB1 were prepared. The protein content of all diets was 51%, the fat content was 9%, and the AFB1 content was 800 µg/kg. The dietary AFB1 concentration was selected based on previous dose–response studies in juvenile hybrid grouper. These studies showed that dietary AFB1 concentrations of 445 μg/kg or higher caused significant growth inhibition, hepatic oxidative stress, inflammatory responses, disturbances in protein and lipid metabolism, and histopathological liver injury [3,23]. Therefore, this study used an AFB1 concentration of 800 μg/kg to establish a model capable of producing measurable physiological and hepatic damage, thereby evaluating the protective effects of different doses of lycopene supplementation. Based on this, lycopene was added at concentrations of 0, 200, 400, 600, and 800 mg/kg, and they were named D0 (control group), D200, D400, D600, and D800, respectively. The AFB1 was procured from Puirui Bang Biology Co., Ltd. (Qingdao, China), and its purified crystals exhibited a purity of >99%. Lycopene, a fat-soluble compound with 10% purity, was acquired from Shengjiade Biology Technology Co., Ltd. (Qufu, China).

The diets were prepared using the following steps: (1) Pre-treatment of lycopene and AFB1. Lycopene was weighed according to the required dosage for each experimental group and fully dissolved in the fish oil of the corresponding experimental diets. Separately, 40 mg of AFB1 was fully dissolved in methanol. This methanolic solution was then transferred into 1 kg of microcrystalline cellulose using a pipette. The original container was rinsed twice with methanol, and the rinse solutions were also added to the cellulose mixture. The mixture was then agitated thoroughly to ensure the AFB1 was uniformly distributed. The mixture was then put into a sealed bag, which was left open in a ventilated, light-protected environment at room temperature to dry completely, yielding a mixture with a concentration of 40 mg AFB1/kg microcrystalline cellulose. Finally, the appropriate quantity of this AFB1-prepared cellulose was weighed out for use in the experimental feed formulations. (2) All feed raw materials were thoroughly ground and then sifted through a 60-mesh screen. The ingredients were precisely weighed according to the feed formulation and mixed thoroughly with the addition of fish oil and water. The mixture was then processed using a twin-screw extruder to form 2.5 mm diameter pellets. To prevent the extruder from overheating during continuous operation, which could affect the stability of lycopene, cool the extruder with ice water after producing each batch of feed and wait until the temperature drops to a safe level before resuming production. Additionally, during the feed mixing stage, use ice water to mix the ingredients to effectively protect the stability of lycopene; this also helps cool the extruder to some extent. These pellets were air-dried in a well-ventilated space until a moisture content of 8–10% was achieved. The finished pellets were sealed and stored at −20 °C.

### 2.3. Management of Fish Farming Trials

The experimental fish were acquired from an aquaculture farm located on Donghai Island (Zhanjiang, China). The rearing experiment was conducted in the indoor aquaculture system at the Marine Biological Research Base of Guangdong Ocean University (Zhanjiang, China). Upon arrival of the juvenile fish, the juvenile fish were acclimated for one week in an outdoor concrete tank measuring 5 m × 4 m × 1.8 m. The experiment commenced only after the fish had completely acclimated to the culture conditions. Select robust and uniformly sized fry with an initial weight of 18.26 ± 0.01 g, randomly divide them into groups. For each treatment (D0, D200, D400, D600, D800), 3 replicates were set up, with each replicate being a 1 m^3^ fiberglass tank, and each tank stocked with 30 fish. Feed the fish twice daily, at 08:00 a.m. and 04:00 p.m. Slowly dispense the feed during each feeding to prevent uneaten feed from spoiling the water quality. Fish should be fed until satiated at each feeding. Record the daily feed intake and mortality per tank; adjust the feed amount based on the groupers actual feeding behavior and current weather conditions. Immediately after the afternoon feeding, use a vacuum cleaner to remove feces from the tanks. Maintain continuous aeration throughout the experiment. Replace 70% of the aquaculture seawater every morning before feeding to ensure good water quality in the tanks. The 8-week experiment was conducted under the following conditions: water temperature 28–30 °C, salinity 26–29, ammonia nitrogen and nitrite concentrations <0.5 mg/L, and dissolved oxygen concentration above 6 mg/L.

### 2.4. Sampling

After the experiment, fast for 24 h before collecting samples. Before sampling, the fish were anesthetized with eugenol (40 mg/L) for subsequent sampling procedures. First, all fish in each tank were weighed and counted to determine growth performance indicators. Next, three fish were randomly selected from each tank, and their body length, body weight, visceral weight, and liver weight were measured for morphological analysis. Six fish were randomly selected from each tank for blood collection. Blood was drawn from the caudal vein using sterile 1 mL syringes and transferred into 1.5 mL centrifuge tubes. After being maintained at 4 °C overnight, the blood samples were centrifuged at 3000 rpm for 15 min at 4 °C. The resulting serum was aliquoted and stored at −80 °C for the determination of serum biochemical parameters and antioxidant enzyme activities. For liver histological examination, liver samples were collected from three fish per tank and fixed in 4% paraformaldehyde at room temperature. These samples were subsequently used for paraffin embedding, hematoxylin and eosin staining, and histopathological observation. For hepatic gene-expression analysis, liver samples were collected from three additional fish per tank, placed in RNase-free tubes containing RNAlater, and stored at −80 °C until RNA extraction and RT-qPCR analysis of antioxidant- and inflammation-related genes. For the determination of hepatic antioxidant capacity, liver samples were collected from three additional fish per tank, placed in 2 mL cryogenic tubes, immediately frozen, and stored at −80 °C until the activities of antioxidant enzymes and related biochemical parameters were measured. For hepatic transcriptome analysis, liver samples were collected from three additional fish per tank, placed in RNase-free cryogenic tubes, immediately frozen, and stored at −80 °C until RNA extraction and transcriptome sequencing. Transcriptome sequencing was subsequently performed using liver samples from the D0 and D200 groups.

### 2.5. Analysis of Basic Dietary Components

The proximate analysis of feed was conducted according to AOAC methods [24]. Moisture content was determined by a constant-weight drying method at 105 °C. The crude protein content was measured using the Kjeldahl method. The crude fat content was determined by Soxhlet extraction, and the crude ash content was quantified through ignition in a muffle furnace at 550 °C.

### 2.6. Biochemical Indicators and Antioxidant Enzymes Activities

Biochemical indicators in serum and liver, along with the activities of antioxidant enzymes, were measured using kits supplied by the Nanjing Jiancheng Bioengineering Research Institute (Nanjing, China) including catalase (CAT), superoxide dismutase (SOD), malondialdehyde (MDA), total antioxidant capacity (T-AOC), alkaline phosphatase (AKP), albumin (ALB), alanine aminotransferase (ALT), aspartate aminotransferase (AST), and total protein (TP). The experimental analysis was performed in strict accordance with the operational procedures specified in the instructions.

### 2.7. Liver Histopathology

The tissue was first fixed in a 4% formalin solution, then dehydrated using a series of ethanol gradients, and finally embedded in paraffin. The embedded liver tissue was then sectioned into 5-micrometer-thick sections, which were deparaffinized with xylene and subsequently immersed sequentially in 100%, 95%, 85% and 75% ethanol solutions for 1 to 5 min each. Next, the sections were rinsed with distilled water for 2 min, followed by staining with hematoxylin for 8 min at room temperature, and then with eosin for 1 min. Observations were conducted using a Leica DM 6000 fluorescence inverted optical microscope (Leica Microsystems, Tokyo, Japan), with images captured using LAS 3.8 software.

### 2.8. Total RNA Extraction, cDNA Synthesis, and Reverse Transcription–Quantitative PCR

Total RNA was extracted from liver samples using the TransZol Up Plus RNA Kit (TransGen Biotech Co., Ltd., Beijing, China) according to the manufacturer’s instructions. First-strand cDNA was synthesized using the PrimeScript™ RT Reagent Kit with gDNA Eraser (Aikerui Bioengineering Co., Ltd., Changsha, China) and stored at −80 °C until analysis.

Reverse transcription–quantitative PCR (RT–qPCR) was performed using a LightCycler^®^ 480 II Real-Time PCR System. Each biological sample was analyzed in three technical replicates. The amplification program consisted of an initial denaturation step at 95 °C for 30 s, followed by 40 cycles of denaturation at 95 °C for 5 s and annealing/extension at 60 °C for 34 s. The primer sequences used for RT–qPCR are listed in Table 2. The *β-actin* gene was used as the reference gene, and the relative mRNA expression levels of the target genes were calculated using the 2^−ΔΔCt^ method [25].

### 2.9. Transcriptome Sequencing and Analysis

Liver transcriptomic sequencing was performed on the control group (D0) and the group with the best growth performance (D200), with three replicates per group. Transcriptome sequencing analysis begins with RNA sample preparation, mRNA enrichment, library construction, and high-throughput sequencing. After obtaining the sequencing data, the first step is to perform data-quality control. Fastp is used to filter the raw reads, removing those containing adapter sequences, those with an excessively high proportion of N bases, all-A sequences, and low-quality reads, resulting in high-quality clean reads. Base quality and composition analyses are performed simultaneously to assess the reliability of the sequencing data. Next, sequence alignment analysis was performed. First, Bowtie2 was used to align the clean reads against an rRNA database to remove any remaining ribosomal RNA reads. Then, HISAT2 was used to align the clean reads against the reference genome. Based on the alignment results, the distribution of reads across exons, introns, and intergenic regions is analyzed to assess the quality of the sequencing data. Using the HISAT2 alignment results, transcripts are reconstructed with StringTie, and Salmon is used to calculate gene or transcript expression levels. Principal component analysis (PCA) was used to examine the overall expression patterns and clustering among samples, and Pearson correlation analysis was used to assess the consistency among biological replicates. The core analysis is the analysis of differentially expressed genes. Using gene read counts as input, perform between-group differential analysis with DESeq2 when biological replicates are present; for two-sample comparisons without biological replicates, use edgeR. After screening for differentially expressed genes, functional enrichment analysis is performed. Based on the results of the differential analysis, we screened for significantly differentially expressed genes using the following criteria: FDR < 0.05 and |log_2_FC| > 1. The sequencing platform used for transcriptomic analysis was the Illumina NovaSeq 6000; the read length was 150 bp paired-end (PE150); and the reference genome assembly version was GCF_005281545.1_ASM528154v1.

### 2.10. Statistical Analysis

The calculation formulas for growth performance and morphological indicators are as follows:

SGR (%/d) = 100% × [(ln (FBW) − ln (IBW)]/days of experiment(1)

SR (%) = 100% × (final number of surviving fish/initial number of fish)(2)

CF (g/cm^3^) = 100% × weight of fish/length of fish^3^ (3)

VSI (%) = 100% × (viscera weight/body weight)(4)

HSI (%) = 100% × (liver weight/body weight)(5)

In the above formulas, FBW, IBW, SGR, SR, CF, VSI, and HIS represent the final body weight (FBW), initial body weight (IBW), Specific growth rate (SGR), Survival rate (SR), Condition factor (CF), Visceralsomatic index (VSI), and Hepatosomatic index (HSI).

The tank was considered the experimental unit because each dietary treatment was administered at the tank level. Therefore, three independent replicate tanks were used for each dietary treatment (*n* = 3 tanks per treatment). Growth performance and survival variables were calculated separately for each tank. For morphological indices, serum biochemical parameters, hepatic antioxidant variables, and RT-qPCR measurements, individual fish sampled from the same tank were considered subsamples rather than independent experimental replicates. The measurements obtained from fish within each tank were averaged to generate one tank-level value before statistical analysis. Thus, all statistical comparisons among dietary treatments were performed using the three tank means for each treatment.

Prior to analysis, the data were tested for normality and homogeneity of variances. Outliers were identified via box plot and IQR method. If outliers occur, we will investigate whether they are caused by non-standard experimental procedures and repeat the analysis. When the data met the criteria for normal distribution and homogeneity of variances, one-way analysis of variance (ANOVA) was performed using SPSS 26.0 software (SPSS Inc., Chicago, IL, USA). When a significant treatment effect was detected, Duncan’s multiple range test was subsequently conducted. Results are presented as mean ± standard error of the mean, calculated from the means of three replicate tanks. Differences were considered statistically significant at *p* < 0.05.

## 3. Results

### 3.1. Growth Performance and Morphological Indicators

**Figure 1:**
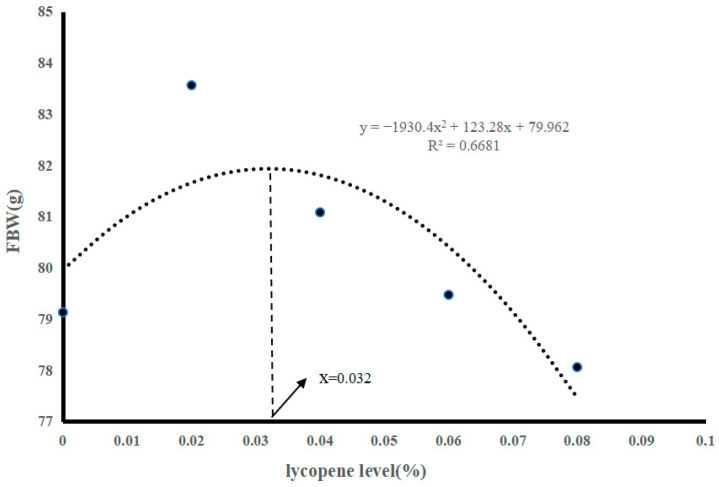
Fitting graph of hybrid grouper FBW versus lycopene supplementation levels.

As shown in Table 3, compared to the D0 group, the D200 group exhibited a significant increase in FBW and SGR (*p* < 0.05). However, as the lycopene content continued to increase, the FBW and SGR of the D400-D800 groups showed a decreasing trend, and there was no significant difference compared to the D0 group (*p* > 0.05). No significant differences in SR were observed among the groups (*p* > 0.05). Morphological indicator analysis found that the addition of lycopene did not have a significant effect on CF, VSI, and HSI (*p* > 0.05). Figure 1 shows a fitted line graph of lycopene levels versus FBW. It can be seen that at a lycopene addition rate of 0.032%, FBW reaches its optimal value.

### 3.2. Serum Antioxidant Capacity and Biochemical Parameters

**Figure 2:**
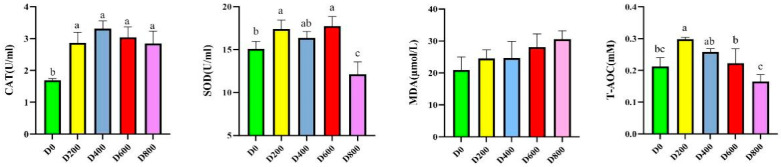
The effect of lycopene supplementation in diet on the serum antioxidant capacity of hybrid grouper induced by AFB1. Values in the figure are means ± SEM (*n* = 3); there are no significant differences between the values bearing the same superscript letter across groups (*p* > 0.05). Catalase (CAT), superoxide dismutase (SOD), malondialdehyde (MDA), total antioxidant capacity (T-AOC). Lycopene was added at concentrations of 0, 200, 400, 600, and 800 mg/kg, and they were named D0, D200, D400, D600, and D800.

As shown in Figure 2, the CAT activity of the D200, D400, D600, and D800 groups were all significantly higher than that of the D0 group (*p* < 0.05), but there were no significant differences among lycopene treated groups (*p* > 0.05). Compared with the D0 group, the SOD activity in the D200 and D600 groups showed a significant increase (*p* < 0.05), while it experienced a sharp decline in the D800 group (*p* < 0.05). No significant differences in MDA content were observed across the groups (*p* > 0.05). The T-AOC of the D200 group was significantly higher than that of the D0, D600, D800 groups, and the D800 group has the minimum value (*p* < 0.05).

**Figure 3:**
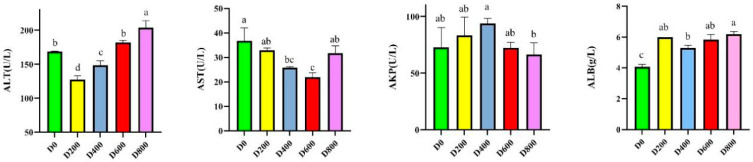
The effects of lycopene supplementation in feed on serum biochemical parameters in hybrid grouper induced by AFB1. Values in the figure are means ± SEM (*n* = 3); there are no significant differences between the values bearing the same superscript letter across groups (*p* > 0.05). Alkaline phosphatase (AKP), albumin (ALB), alanine aminotransferase (ALT), aspartate aminotransferase (AST). Lycopene was added at concentrations of 0, 200, 400, 600, and 800 mg/kg, and they were named D0, D200, D400, D600, and D800.

Figure 3 illustrates that the D200 group exhibited a significantly lower ALT activity compared to the D0 group and all other groups (*p* < 0.05). The AST activity reached its minimum in the D600 group, whereas both ALT and AST exhibited a biphasic trend, initially decreasing and subsequently increasing. Compared with the control group on D0, the addition of lycopene did not cause a significant change in AKP activity in all treatment groups (*p* > 0.05). In comparison to the D0 group, the D200-D800 groups exhibited a significant elevation in ALB (*p* < 0.05).

### 3.3. Histomorphology of Liver

**Figure 4:**
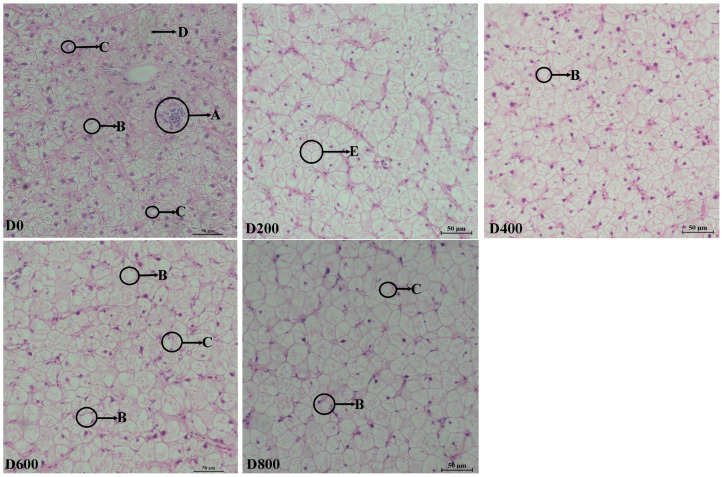
The effects of lycopene on the histological structure of AFB1-induced hybrid grouper liver tissue (H&E, 400×). Notes: A, inflammatory cell infiltration; B, hepatocyte vacuolization; C, Steatosis; D, nucleus; E, cell contour lycopene was added at concentrations of 0, 200, 400, 600, and 800 mg/kg, and they were named D0, D200, D400, D600, and D800.

Figure 4 shows the effects of adding different concentrations of lycopene to the diet on the histology of hybrid grouper livers under AFB1-induced conditions. The D0 group, which received no lycopene, exhibited hepatocyte vacuolization, steatosis, and inflammatory cell infiltration. Compared with the D0 group, the D200 group showed reduced pathological damage to the liver. Hepatocytes had well-defined contours and were tightly packed, with no obvious inflammation or lipid deposition observed. However, as the amount of lycopene supplementation increased further, its efficacy in maintaining the morphological integrity of liver tissue diminished, leading to the reappearance of hepatocyte vacuolization and steatosis in the D600 and D800 groups.

### 3.4. Liver Antioxidant Enzymes Activities

**Figure 5:**
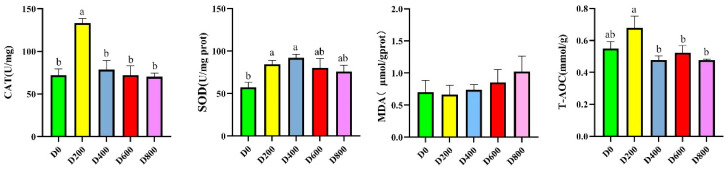
The effects of lycopene on hepatic antioxidant enzymes activities of the liver in hybrid grouper induced by AFB1. Values in the figure are means ± SEM (*n* = 3); there are no significant differences between the values bearing the same superscript letter across groups (*p* > 0.05). catalase (CAT), superoxide dismutase (SOD), malondialdehyde (MDA), total antioxidant capacity (T-AOC). Lycopene was added at concentrations of 0, 200, 400, 600, and 800 mg/kg, and they were named D0, D200, D400, D600, and D800.

As shown in Figure 5, the CAT activity in the liver was significantly higher in the D200 group than in the D0, D400, D600, and D800 groups (*p* < 0.05). Compared to the D0 group, the SOD activity in the liver of the D200 and D400 groups was significantly elevated (*p* < 0.05), whereas in the D600 and D800 groups it did not differ significantly from the D0 group (*p* > 0.05). The MDA content in livers of each group showed no significant difference (*p* > 0.05), and the T-AOC level in each treatment group showed no significant difference compared with the control group D0 *(p* > 0.05). However, the overall trend showed an initial increase followed by a decrease.

### 3.5. Gene-Expression Levels in the Liver

**Figure 6:**
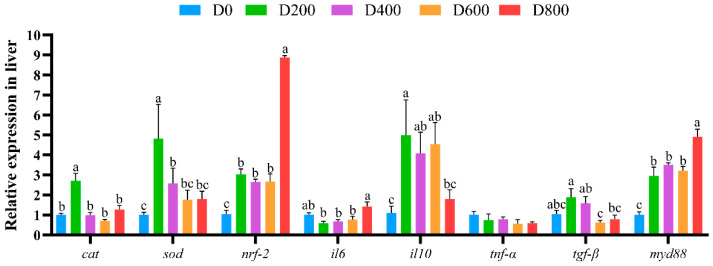
Effects of lycopene on antioxidant activity and inflammatory cytokine gene expression in the liver of hybrid grouper induced by AFB1. Values in the figure are means ± SEM (*n* = 3); there are no significant differences between the values bearing the same superscript letter across groups (*p* > 0.05)*. cat* (catalase), *sod* (superoxide dismutase), *nrf-2* (nuclear factor E2related factor 2), *il6* (interleukin 6), *il10* (interleukin 10), tnf-α (tumor necrosis factor-α), *tgf-β* (transforming growth factor beta), *myd88* (myeloid differentiation primary response 88).

As shown in Figure 6, compared with D0 group, the addition of 200 mg/kg lycopene significantly increased the gene-expression levels of *cat*, *sod*, and *nrf-2* in antioxidant genes (*p* < 0.05). In the gene-expression levels of inflammatory factors, the addition of lycopene significantly upregulated the expression of the anti-inflammatory factor *il10* and the maximum value was found in group D2 (*p* < 0.05). Although the addition of lycopene did not show a significant change in the expression level of *tgf-β*, it showed an overall trend of first rising and then falling. Compared to the D0 group, the expression levels of the pro-inflammatory factors *il6* and *tnf-α* in lycopene treated groups did not show significant changes, but the gene-expression level of *il6* showed a trend of first decreasing and then increasing, while the gene-expression level of *tnf-α* showed a decreasing trend with increasing lycopene dosage. Supplementation with lycopene resulted in a significant upregulation of *myd88* gene expression (*p* < 0.05).

### 3.6. Hepatic Transcriptome Analysis

#### 3.6.1. Sequencing Quality Assessment

Table 4 presents data on the quality of transcriptomic sequencing. A total of six liver RNA-seq libraries were constructed, with three biological replicates each for the D0 and D200 groups. The sequencing depth ranged from 39.60 to 50.24 million clean reads per library, with an average of approximately 45.93 million clean reads. The Q20 and Q30 values ranged from 98.64% to 98.87% and from 95.37% to 96.12%, respectively, while the GC contents were consistently distributed between 49.48% and 49.83%. The overall mapping rates to the reference genome ranged from 74.84% to 77.12%. The relatively small variation in sequencing quality, base composition, and mapping efficiency among the libraries indicated that the transcriptomic data were stable and suitable for subsequent differential gene-expression and functional enrichment analyses.

#### 3.6.2. Identification of DEGs

**Figure 7:**
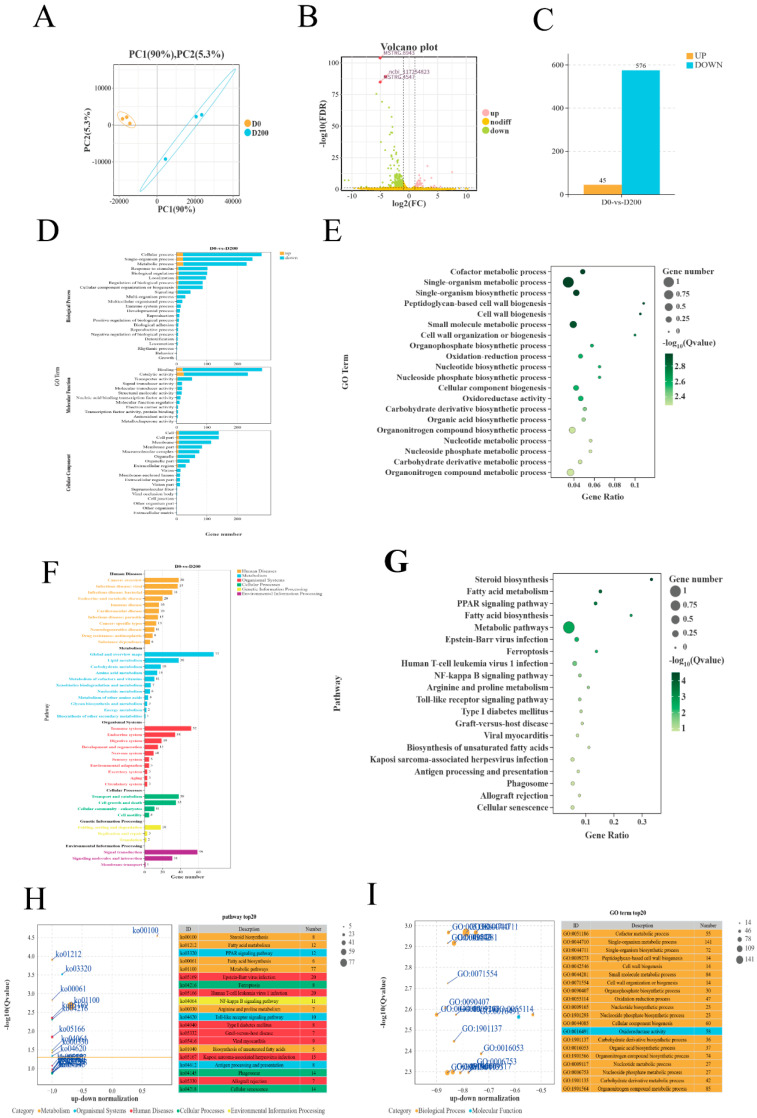
The effects of lycopene on hepatic transcriptome of the liver in hybrid grouper induced by AFB1. Notes: (**A**): Principal Component Analysis (PCA). (**B**): Volcano Plot of Differentially Expressed Genes. (**C**): Statistics on the Number of Differentially Expressed Genes. (**D**): GO Feature Categories. (**E**): GO Enrichment Analysis Bubble Chart. (**F**): KEGG Pathway Categories. (**G**): KEGG Pathway Enrichment Analysis Bubble Chart. (**H**): Expression Direction and Significance of the Top 20 KEGG Pathways. (**I**): Expression Directions and Significance of the Top 20 GO Terms.

Figure 7 presents the liver transcriptome analysis of the hybrid grouper. Principal component analysis showed a clear separation between the D0 and D200 groups, with PC1 and PC2 explaining 90.0% and 5.3% of the total variance, respectively, indicating distinct hepatic transcriptional profiles between the two treatments (Figure 7A). A total of 621 differentially expressed genes were identified between the D0 and D200 groups (Figure 7B,C).

#### 3.6.3. Enrichment Analysis of Differentially Expressed Genes (DEGs)

Functional enrichment analysis showed that the transcriptional differences were mainly associated with hepatic metabolism and redox regulation. The major enriched GO terms included small-molecule metabolism, organic acid metabolism, organonitrogen compound metabolism, oxidoreductase activity, and oxidation–reduction processes (Figure 7D,E). KEGG enrichment analysis further highlighted pathways relevant to AFB1-associated metabolic disturbance and the potential protective response to lycopene, particularly fatty acid metabolism, fatty acid biosynthesis, biosynthesis of unsaturated fatty acids, steroid biosynthesis, and the PPAR signaling pathway (Figure 7F–H).

Ferroptosis and inflammation- and innate-immunity-related pathways, including NF-κB and Toll-like receptor signaling, were also enriched. Collectively, these results indicate that the hepatic response to 200 mg/kg lycopene under AFB1 exposure was primarily associated with the regulation of lipid metabolism and redox homeostasis, with additional involvement of lipid-peroxidation-related cell injury and immune signaling.

## 4. Discussion

Aflatoxin B1 (AFB1) is a mycotoxin of significant concern in aquafeeds. This is particularly pertinent given the increasing proportion of plant-based ingredients in grouper feeds, which elevates the risk of contamination and warrants greater attention. Studies on hybrid grouper have indicated that exposure to AFB1 can lead to stunted growth, liver tissue damage, and can disrupt metabolic pathways—including cytochrome P450 metabolism and the biosynthesis of unsaturated fatty acids. AFB1 can also induce elevated levels of ROS and MDA while reducing the activity of antioxidant enzymes, including SOD, CAT, and GST [3,23]. As a lipid-soluble carotenoid, lycopene functions to scavenge free radicals, enhance the activity of endogenous antioxidant enzymes, modulate the Nrf2-Keap1 pathway, suppress inflammation, and ameliorate mitochondrial function [19,20,26,27]. Its protective effect has been demonstrated in various animal models [28].

The results of this experiment indicate that supplementing the diet with 200 mg/kg of lycopene mitigates the growth impairment induced by AFB1, leading to significant improvements in FBW and SGR; this is consistent with the findings from prior research on aquatic species including *Ctenopharyngodon idellus*, *Oncorhynchus mykiss*, and *Oreochromis niloticus* [29,30,31]. Although the experimental conditions, fish species, and toxic stressors varied, lycopene at an appropriate concentration consistently demonstrated a growth-promoting effect across all scenarios. Notably, no significant differences were observed among the groups for the following indicators: SR, CF, VSI, and HSI. This suggests that the primary beneficial effect of lycopene on hybrid grouper exposed to AFB1 stress is not manifested in terms of survival rates or morphological metrics; rather, it is more prominently manifested in SGR, antioxidant capacity, and the status of liver function.

Oxidative stress is a major cause of AFB1-induced hepatotoxicity. Previous research in juvenile hybrid grouper showed that dietary AFB1 exposure increased hepatic reactive oxygen species production, weakened antioxidant defense, and induced inflammatory and metabolic disturbances [3]. As key enzymatic antioxidants, SOD and CAT cooperatively maintain cellular redox homeostasis: SOD eliminates superoxide anions to generate H_2_O_2_, while CAT further decomposes H_2_O_2_ to reduce ROS-induced oxidative damage to cellular biomacromolecules [32,33,34,35]. Owing to its conjugated double-bond structure, lycopene effectively scavenges free radicals and relieves oxidative stress. Consistent with previous studies in broilers, lambs, *Oreochromis niloticus* and *Oncorhynchus mykiss*, moderate lycopene supplementation can markedly improve hepatic antioxidant capacity under multiple stress conditions [36,37,38,39]. In this study, serum and hepatic antioxidant indices presented consistent improvements with lycopene supplementation. The D200 group exhibited optimal serum CAT, SOD and T-AOC levels, as well as the highest hepatic CAT activity. Additionally, both the D200 and D400 groups showed significantly higher hepatic SOD activity than the D0 group. Collectively, these results indicate that appropriate lycopene supplementation efficiently enhances systemic and hepatic antioxidant defense in AFB1-exposed hybrid grouper.

ALT is predominantly localized in the hepatocyte cytoplasm and is released into the blood upon hepatocyte damage induced by inflammation, toxins, or metabolic dysfunction. Accordingly, elevated serum ALT activity serves as a reliable biomarker of liver injury [40,41]. Hepatocyte injury or increased membrane permeability can result in the release of AST into the blood, causing elevated serum AST activity. Consequently, AST is also considered an indicator for assessing liver damage [42]. The ALT levels in the D200 group were significantly lower than in the D0 group, suggesting that a moderate dose of lycopene can mitigate hepatocyte damage induced by AFB1. These findings are consistent with the results of prior research conducted on carp [30]. The D800 group exhibited the highest ALT, indicating that a high dose of lycopene did not continue to provide a protective effect and may have even exacerbated oxidative damage to hepatocytes. AST was lowest in the D600 group, but due to its broader tissue sources in fish, AST is less specific than ALT. AKP is a key enzymatic marker for assessing liver and biliary function as well as bone metabolism. This enzyme is highly concentrated in the liver and skeletal tissues. Consequently, serum AKP levels can markedly increase in cases of liver disease, bile duct damage, or disorders of hepato-biliary excretion. In this study, no significant differences in AKP activity were observed between the treatment groups and the D0 control group. Almroth et al.’s study found that excessively fast growth rates lead to elevated enzyme levels within the body of *Oncorhynchus kisutch* [43]. Consequently, the AKP level in the D200 and D400 groups exhibited an increasing trend in this experiment, a phenomenon potentially attributable to elevated AKP levels resulting from the accelerated growth rate of the fish. ALB is the principal plasma protein synthesized by the liver, responsible for maintaining plasma colloid osmotic pressure and transporting a wide variety of endogenous and exogenous substances. Serum ALB level can reflect liver protein synthesis function and the body’s nutritional status, and its decrease usually indicates impaired liver synthesis function and/or malnutrition [44,45]. In the group supplemented with lycopene, ALB levels were higher than those in the D0 group, indicating that lycopene supplementation, under AFB1 induction, promoted protein synthesis and nutrient metabolism.

The liver serves as the principal organ for metabolism, with histological changes providing a direct reflection of its health condition. The D0 group exhibited inflammatory cell infiltration, hepatocyte vacuolation, fatty degeneration, and nuclear abnormalities, all signs of significant liver damage induced by AFB1. Upon the addition of lycopene, there was observed improvement in hepatocyte morphology, tissue architecture, and the severity of pathological damage, demonstrating its potential to mitigate the structural deterioration of liver tissue caused by AFB1. Previous research on hybrid grouper has shown that AFB1 exposure results in several pathological alterations in liver tissue, including inflammation, karyorrhexis, pyknosis, karyolysis, focal necrosis, and ballooning degeneration [3], this is consistent with the liver cell vacuolization, steatosis, and inflammatory response observed in the D0 group of this study. This also indicates that in the assessment of fish health, histopathological and micro-biochemical markers are more sensitive to change than macro-morphological markers [46].

Both the *cat* and *sod* genes are closely associated with antioxidant defense; together, they help eliminate excess reactive oxygen species in the body, reduce oxidative stress levels, protect liver cells from free radical damage, and maintain intracellular redox balance [47]. *nrf-2* is an important transcriptional regulator of genes involved in antioxidant responses and can regulate the expression of various genes associated with oxidative damage, inflammation, and the free radical response [48]. The concurrent upregulation of *nrf-2*, *cat*, and *sod* observed in this study suggests that the improvement in liver antioxidant capacity induced by lycopene may be related to the activation of the Nrf2 signaling pathway. This finding is generally consistent with studies in other species. In broiler chickens research, the addition of lycopene to the diet increases antioxidant markers such as SOD and GSH-Px in serum and liver, and upregulates genes related to the Keap1-Nrf2 pathway—including *nrf-2* and *sod* in the liver [36]. In a study by Wang et al. [36], lycopene was found to alleviate pollutant-induced oxidative stress, inflammation, and apoptosis by regulating NF-κB balance. Therefore, the upregulation of *cat*, *sod*, and *nrf-2* in the livers of hybrid grouper in this study is consistent with findings showing that lycopene enhances antioxidant defenses in poultry and other fish species. Important pro-inflammatory cytokines in the inflammatory response are typically *il6* and *tnf-α*, and *tnf-α* also plays a significant role in the early immune response to infection in fish [49,50,51]. Therefore, the fact that pro-inflammatory genes were not significantly activated after lycopene treatment and that *tnf-α* levels, in particular, showed a downward trend suggests that lycopene may maintain hepatic immune homeostasis by reducing oxidative stress levels, thereby indirectly inhibiting the excessive activation of inflammatory pathways such as NF-κB. *il10* is generally considered an important anti-inflammatory cytokine that can limit excessive inflammatory responses [52]. Moreover, *tgf-β* is involved in maintaining immune homeostasis, suppressing inflammation, and facilitating tissue repair [53]. In the experimental results, the increase in *il10* in the 200 mg/kg lycopene group may indicate that an appropriate dose of lycopene not only enhances antioxidant defense but also promotes the regulation of the liver inflammatory response towards an anti-inflammatory and reparative direction. Similarly, in an AFB1-exposed chick intestinal model, lycopene can inhibit the increase in inflammatory factors induced by AFB1, and increase *il10* levels, improving intestinal barrier and immune function [27].

It is worth noting that when dietary lycopene supplementation exceeded 200 mg/kg, both growth performance and antioxidant capacity declined. This phenomenon may be attributed to lycopene’s dual antioxidant and pro-oxidant properties: its conjugated double bonds, which are responsible for scavenging free radicals, are depleted upon excessive intake, thereby generating lycopene radical intermediates and leading to increased accumulation of ROS [54]. As a fat-soluble carotenoid primarily deposited in the liver and adipose tissue [55], excessive lycopene accumulates in fish hepatocytes, leading to hepatic lipid deposition and liver dysfunction. Furthermore, while an appropriate amount of lycopene activates the TOR pathway to promote protein synthesis, excessive lycopene induces oxidative stress, inhibits the PI3K-AKT-TOR pathway, impedes protein deposition, and reduces fish growth performance [56]. This mechanism provides a sound explanation for the reduced growth performance, weakened antioxidant capacity, and exacerbated hepatic morphological damage observed in hybrid grouper fed high doses of lycopene in this study.

Principal component analysis showed a clear separation between the D0 and D200 groups, indicating that dietary lycopene supplementation substantially altered the hepatic transcriptional response of hybrid grouper under AFB1 exposure. A total of 621 differentially expressed genes were identified between the two groups, and these genes were mainly associated with oxidation–reduction processes, fatty acid metabolism, the biosynthesis of unsaturated fatty acids, the PPAR signaling pathway, ferroptosis, and inflammation- and innate-immunity-related pathways. In a study of *Acanthopagrus schlegelii* [57], the results of GO enrichment analysis of the liver transcriptome following the addition of lycopene under a high-carbohydrate diet were generally consistent with the results of this experiment. These biological processes are closely related to the major features of AFB1 hepatotoxicity, including oxidative stress, lipid metabolic disturbance, lipid peroxidation, inflammation, and hepatocellular injury. Therefore, the transcriptomic responses were generally consistent with the biochemical, gene-expression, and histological changes observed in the D200 group.

The enrichment of oxidation–reduction-related DEGs may partly explain the improvement in antioxidant capacity following lycopene supplementation. Compared with the D0 group, the D200 group showed increased serum and hepatic CAT and SOD activities, together with improved serum T-AOC. In parallel, the hepatic expression levels of *nrf-2*, cat, and sod were significantly upregulated. *Nrf-2* is an important regulator of cellular redox homeostasis and promotes the expression of antioxidant enzymes responsible for the elimination of reactive oxygen species. The synchronous changes in the redox-related transcriptome, antioxidant gene expression, and enzyme activity indicate that lycopene enhanced the in vivo antioxidant capacity of hybrid grouper exposed to AFB1 and alleviated oxidative damage to hepatocytes. This is consistent with the observations of reduced serum ALT activity and decreased hepatic vacuolization in the D200 group.

The DEGs were enriched in fatty acid metabolism, the biosynthesis of unsaturated fatty acids, and the PPAR signaling pathway. PPAR-related signaling plays an important role in fatty acid transport, lipid utilization, and mitochondrial β-oxidation [58]. Disturbance of these processes during AFB1 exposure may promote abnormal lipid deposition and hepatic steatosis. Previous studies of hybrid grouper [23] have shown that AFB1 exposure impairs lipid metabolism and induces hepatic pathological changes, including steatosis. PPAR signaling is a central regulatory pathway for fatty acid uptake, β-oxidation, lipid storage, and energy homeostasis [59]. In a study by Wang et al. [60], lycopene activated the PPARα signaling pathway in the liver, upregulated the expression of key genes involved in fatty acid transport and oxidation, inhibited excessive lipid synthesis and accumulation in hepatocytes, and improved mitochondrial dysfunction and lipid metabolism disorders induced by exogenous stimuli such as palmitic acid and mycotoxins. In the study, the reduction in hepatic steatosis in the D200 group was consistent with the transcriptional changes in lipid metabolism-related pathways, suggesting that lycopene may help maintain hepatic lipid homeostasis by regulating fatty acid utilization and metabolism.

The enrichment of NF-κB and Toll-like receptor signaling pathways further indicates that oxidative stress can stimulate Toll-like receptor-associated signaling and promote NF-κB-mediated inflammatory responses, thereby aggravating hepatic injury. In the present study, the increased expression of the anti-inflammatory cytokine *il10* and the reduced inflammatory cell infiltration in the D200 group were consistent with improved hepatic immune homeostasis. Changes in *myd88* expression also support the involvement of Toll-like receptor-associated signaling. However, pathway enrichment alone cannot establish whether the entire Toll-like receptor or NF-κB pathway was activated or suppressed. Thus, lycopene should be considered to have modulated inflammation-related signaling rather than to have completely inhibited the inflammatory response.

Taken together, the physiological, biochemical, histological, gene-expression, and transcriptomic results consistently indicate that 200 mg/kg lycopene is the most effective supplementation level for mitigating AFB1-induced impairment in hybrid grouper under the present experimental conditions. The protective effects appear to be associated with enhancement of antioxidant defenses, improvement of hepatic structural integrity, modulation of lipid metabolism, and regulation of redox and immune related pathways.

This study also has several limitations. First, due to the absence of an AFB1-free control group, it was not possible to determine whether the extent of tissue damage repair mediated by lycopene restored physiological levels comparable to those observed in fish unexposed to AFB1. Transcriptomic sequencing was only performed on the D0 and D200 groups; consequently, the dose-dependent molecular responses induced by high-dose lycopene supplementation could not be evaluated. Second, although pathway enrichment analysis revealed associations with relevant biological processes, it could not independently verify the activation or inhibition of specific signaling pathways. In subsequent related experiments, we will include control groups devoid of AFB1 and lycopene, and further validate representative genes and proteins involved in fatty acid metabolism, PPAR signaling, ferroptosis, and inflammatory responses.

## 5. Conclusions

In conclusion, dietary supplementation with 200 mg/kg lycopene effectively alleviated AFB1-induced growth retardation and liver injury in hybrid grouper. This protective effect was reflected by improved FBW and SGR, enhanced serum and hepatic antioxidant capacity, decreased serum ALT activity, increased ALB level, improved liver histology, and upregulated antioxidant-related gene expression. Transcriptomic analysis further indicated that the protective action of lycopene was closely associated with the regulation of metabolic pathways, fatty acid metabolism, PPAR signaling, oxidation–reduction processes, ferroptosis, and immune-related pathways. Based on the fitted quadratic regression graph, the recommended lycopene addition level is 0.032%. Under the present experimental conditions, 200 mg/kg lycopene was the optimal supplemental level, whereas higher levels did not produce additional benefits.

## Figures and Tables

**Table 1:** Feed formula and nutrient composition (dry matter %).

Ingredients	Groups				
	D0	D200	D400	D600	D800
Fish meal	43	43	43	43	43
Chicken meal	10	10	10	10	10
Wheat gluten	6	6	6	6	6
Soy protein concentrate	13	13	13	13	13
Wheat flour	16	16	16	16	16
Phospholipid	1.5	1.5	1.5	1.5	1.5
Fish oil	3	3	3	3	3
Compound premix ^a^	1	1	1	1	1
Choline chloride	0.5	0.5	0.5	0.5	0.5
Ca(H_2_PO_4_)_2_	1	1	1	1	1
Microcrystalline Cellulose	5	4.98	4.96	4,94	4.92
Lycopene	0	0.02	0.04	0.06	0.08
AFB1 (µgkg^−1^) ^c^	800	800	800	800	800
Total	100	100	100	100	100
Proximate composition ^b^					
Crude protein	51.27	51.36	51.73	51.04	51.42
Crude lipid	9.52	9.65	9.16	9.51	9.25
Ash	11.93	11.93	11.86	11.92	11.97
T-AOC (mmol/g)	0.27	0.32	0.35	0.39	0.45

^a^ Compound premix was obtained from Qingdao Master Biotechnology Co., Ltd. (Qingdao, China) Vitamin and Mineral Premix (kg^−1^ of diet): thiamine, 5 mg; riboflavin, 10 mg; vitamin A, 5000 IU; vitamin E, 40 mg; vitamin D3, 1000 IU; menadione, 10 mg; pyridoxine, 10 mg; biotin, 0.1 mg; cyanocobalamin, 0.02 mg; calcium pantothenate, 20 mg; folic acid, 1 mg; niacin, 40 mg; vitamin C, 150 mg; iron, 100 mg; iodine, 0.8 mg; cupper, 3 mg; zinc, 50 mg; manganese, 12 mg; selenium, 0.3 mg; cobalt, 0.2 mg. ^b^ These values represent the approximate chemical analysis of the diet. ^c^ Aflatoxin B1.

**Table 2:** Primer sequences of target genes used for RT-qPCR analysis.

Primers Names	Primer Sequence (5′ to 3′)	NCBI Accession No.
*β-actin*-F/R	GGCTACTCCTTCACCACCACA/	XM-033645256.1
	TCTGGGCAACGGAACCTCT	
*cat*-F/R	CGCGGGAAGCAAAGATTCAG/	XM-033637630.1
	CCGCAGTTTCCAGTGTGTTG	
*sod*-F/R	CTACTGATGCGGACAGGCAT/	XM-049593128.1
	CCCCCTTTTCCCAGGTCATC	
*nrf-2*-F/R	TATGGAGATGGGTCCTTTGGTG/	XM-033637210.1
	GCTTCTTTTCCTGCGTCTGTTG	
*il6*-F/R	ATGCCCTCTACACTCAACGC/	XM-033638018.2
	TCAAACTGCTTTTCGTGGCG	
*il10*-F/R	CCATTGCTGTCGATTCGTGG/	XM-033625798.2
	GCAGGCAAACGGGCTTTTA	
*tnf-α*-F/R	GCAGCGATGGTGACGAGAAGG/	XM-033640148.1
	TCCTCCTGTGCCGTGCTCTG	
*tgf-β*-F/R	TGGCTCAAAGGGAATGAGGAT/	XM-033631740.2
	AGTTGCCTCACAACCGAACAC	
*myd88*-F/R	GCCAGAGCGACTTTGAGTTTG/	XM-033638920.2
	ACCATCCGCTTACACCTCTTCT	

Notes: *β-actin* (beta actin), *cat* (catalase), *sod* (superoxide dismutase), *nrf-2* (nuclear factor E2related factor 2), *il6* (interleukin 6), *il10* (interleukin 10), tnf-α (tumor necrosis factor-α), *tgf-β* (transforming growth factor beta), *myd88* (myeloid differentiation primary response 88).

**Table 3:** The effects of lycopene on the growth performance and morphology indicators of hybrid grouper under the influence of AFB1.

Parameters	Groups				
	D0	D200	D400	D600	D800
IBW (g)	18.21 ± 0.01	18.21 ± 0.03	18.27 ± 0.02	18.28 ± 0.03	18.26 ± 0.01
FBW (g)	79.13 ± 1.44 ^b^	83.56 ± 0.29 ^a^	81.08 ± 0.81 ^b^	79.47 ± 1.36 ^b^	78.06 ± 0.56 ^b^
SGR (%/d)	3.12 ± 0.04 ^b^	3.24 ± 0.01 ^a^	3.13 ± 0.01 ^b^	3.13 ± 0.03 ^b^	3.09 ± 0.02 ^b^
SR (%)	97.78 ± 0.02	94.44 ± 0.02	98.89 ± 0.01	97.78 ± 0.01	96.67 ± 0.01
CF (g/cm^3^)	2.55 ± 0.12	2.85 ± 0.13	2.65 ± 0.08	2.75 ± 0.05	2.77 ± 0.12
VSI (%)	8.17 ± 0.51	7.56 ± 0.43	7.46 ± 0.54	8.06 ± 0.16	7.54 ± 0.24
HSI (%)	1.59 ± 0.15	1.30 ± 0.12	1.31 ± 0.19	1.53 ± 0.13	1.38 ± 0.14

Notes: Values in the table are means ± SEM (*n* = 3); values in the same row with the same superscript letter or absence of superscripts are not significantly different (*p* > 0.05); different lowercase letters indicate a significant difference (*p* < 0.05). Initial body weight (IBW), final body weight (FBW), specific growth rate (SGR), survival rate (SR), condition factor (CF), visceralsomatic index (VSI), hepatosomatic index (HSI).

**Table 4:** Summary statistics of liver transcriptome sequencing data.

Sample	Sequencing Depth	Mapping Rate (%)	GC (%)	Q20 (%)	Q30 (%)
D0-1	49,311,654	77.12	49.65	98.75	95.67
D0-2	50,238,446	77.07	49.48	98.87	96.12
D0-3	46,555,458	76.40	49.62	98.64	95.37
D200-1	48,253,228	76.09	49.55	98.86	96.07
D200-2	39,596,478	75.09	49.83	98.83	95.99
D200-3	41,626,490	74.84	49.83	98.79	95.86

## Data Availability

The data supporting the findings of this study are available upon request from the corresponding author. Due to privacy or ethical restrictions, these data are not publicly available.

## References

[1] Li S., Li Z., Sang C., Zhang J., Chen N., Huang X. (2018). Glucose Transporters in Pearl Gentian Grouper (*Epinephelus fuscoguttatus* ♀ × *E. lanceolatus* ♂): Molecular Cloning, Characterization, Tissue Distribution and Their Expressions in Response to Dietary Carbohydrate Level. Aquac. Res..

[2] Das S.K., Xiang T.W., Noor M.N., De M., Mazumder S.K., Goutham-Bharathi M.P. (2021). Temperature Physiology in Grouper (Epinephelinae: Serranidae) Aquaculture: A Brief Review. Aquac. Rep..

[3] Liu H., Liang S., Huang W., Yang Y., Zhou M., Lu B., Li B., Cai W., Song H., Tan B. (2024). Effects of Aflatoxin B1 on Subacute Exposure of Hybrid Groupers (*Epinephelus fuscoguttatus* ♀ × *Epinephelus lanceolatus* ♂): Growth, Liver Histology, and Integrated Liver Transcriptome and Metabolome Analysis. Anim. Nutr..

[4] Tacon A.G.J., Metian M. (2008). Global Overview on the Use of Fish Meal and Fish Oil in Industrially Compounded Aquafeeds: Trends and Future Prospects. Aquaculture.

[5] Gruber-Dorninger C., Mueller A., Rosen R. (2025). Multi-Mycotoxin Contamination of Aquaculture Feed: A Global Survey. Toxins.

[6] Akbar A., Majeed F.A., Sadiq M.B., Khan S.A., Rabaan A.A. (2022). Mycotoxins Occurrence in Food Commodities, Their Associated Hazards and Control Strategies. J. Hell. Vet. Med. Soc..

[7] Gourd E. (2023). High Concentrations of Aflatoxin in Ugandan Grains Sparks Public Health Concern. Lancet Oncol..

[8] Kutasi K., Recek N., Zaplotnik R., Mozetic M., Krajnc M., Gselman P., Primc G. (2021). Approaches to Inactivating Aflatoxins-A Review and Challenges. Int. J. Mol. Sci..

[9] Umar A., Qanita S., Zada N., Honey S.F. (2025). From Feed to Food—Understanding the Impact of Aflatoxins Consumption by Pakistani Livestock. CABI One Health.

[10] Wan X., Ji H., Ma H., Yang Z., Li N., Chen X., Chen Y., Yang H., Wang Z. (2022). Lycopene Alleviates Aflatoxin B1 Induced Liver Damage through Inhibiting Cytochrome 450 Isozymes and Improving Detoxification and Antioxidant Systems in Broiler Chickens. Ital. J. Anim. Sci..

[11] Wan X.L., Li N., Chen Y.J., Chen X.S., Yang Z., Xu L., Yang H.M., Wang Z.Y. (2021). Protective Effects of Lycopene on Mitochondrial Oxidative Injury and Dysfunction in the Liver of Aflatoxin B1-Exposed Broilers. Poult. Sci..

[12] Liu Y., Wang W. (2016). Aflatoxin B1 Impairs Mitochondrial Functions, Activates ROS Generation, Induces Apoptosis and Involves Nrf2 Signal Pathway in Primary Broiler Hepatocytes. Anim. Sci. J..

[13] Anater A., Manyes L., Meca G., Ferrer E., Luciano F.B., Pimpao C.T., Font G. (2016). Mycotoxins and Their Consequences in Aquaculture: A Review. Aquaculture.

[14] El-Sayed Y.S., Khalil R.H. (2009). Toxicity, Biochemical Effects and Residue of Aflatoxin B1 in Marine Water-Reared Sea Bass (*Dicentrarchus labrax* L.). Food Chem. Toxicol..

[15] Liu H., Xie R., Huang W., Yang Y., Zhou M., Lu B., Li B., Tan B., Dong X. (2024). Effects of Dietary Aflatoxin B1 on Hybrid Grouper (*Epinephelus fuscoguttatus* ♀ × *Epinephelus lanceolatus* ♂) Growth, Intestinal Health, and Muscle Quality. Aquac. Nutr..

[16] Moloi T.P., Ziqubu K., Mazibuko-Mbeje S.E., Mabaso N.H., Ndlovu Z. (2024). Aflatoxin B1-Induced Hepatotoxicity through Mitochondrial Dysfunction, Oxidative Stress, and Inflammation as Central Pathological Mechanisms: A Review of Experimental Evidence. Toxicology.

[17] Cao L., Zhao J., Ma L., Chen J., Xu J., Rahman S.U., Feng S., Li Y., Wu J., Wang X. (2021). Lycopene Attenuates Zearalenone-Induced Oxidative Damage of Piglet Sertoli Cells through the Nuclear Factor Erythroid-2 Related Factor 2 Signaling Pathway. Ecotox. Environ. Saf..

[18] Fallah R., Kiani A., Khaldari M. (2021). Supplementing Lycopene Combined with Corn Improves Circulating IgG Concentration in Pregnant Ewes and Their Lambs. Trop. Anim. Health Prod..

[19] Huang W., Cao Z., Cui Y., Huo S., Shao B., Song M., Cheng P., Li Y. (2023). Lycopene Ameliorates Aflatoxin B1-Induced Testicular Lesion by Attenuating Oxidative Stress and Mitochondrial Damage with Nrf2 Activation in Mice. Ecotox. Environ. Saf..

[20] Zhao H., Wang Y., Mu M., Guo M., Yu H., Xing M. (2020). Lycopene Alleviates Sulfamethoxazole-Induced Hepatotoxicity in Grass Carp (*Ctenopharyngodon idellus*) Via suppression of Oxidative Stress, Inflammation and Apoptosis. Food Funct..

[21] Zhu C., Song Z., Ye Z., Zhang Y., Liu J., Xiao L., Bian C., Ma Q., Wei Y., Liang M. (2026). Dietary Lysophospholipids Enhance the Function of Lutein Rather than Lycopene in Pacific White Shrimp. Fishes.

[22] Mekkawy I.A.A., Mahmoud U.M., Wassif E.T., Naguib M. (2011). Effects of Cadmium on Some Haematological and Biochemical Characteristics of *Oreochromis niloticus* (Linnaeus, 1758) Dietary Supplemented with Tomato Paste and Vitamin E. Fish Physiol. Biochem..

[23] Liu H., Xie R., Huang W., Yang Y., Zhou M., Lu B., Li B., Tan B., Dong X. (2023). Negative Effects of Aflatoxin B1 (AFB1) in the Diet on Growth Performance, Protein and Lipid Metabolism, and Liver Health of Juvenile Hybrid Grouper (*Epinephelus fuscoguttatus* ♀ × *Epinephelus lanceolatus* ♂). Aquac. Rep..

[24] Feldsine P., Abeyta C., Andrews W.H. (2002). AOAC INTERNATIONAL Methods Committee Guidelines for Validation of Qualitative and Quantitative Food Microbiological Official Methods of Analysis. J. AOAC Int..

[25] Livak K.J., Schmittgen T.D. (2001). Analysis of Relative Gene Expression Data Using Real-Time Quantitative PCR and the 2^−ΔΔCT^ Method. Methods.

[26] Lu J., Zhou L., Liu C., Qian H. (2025). Lycopene Modulates Glycerophospholipid Metabolism and Promotes N-Acylethanolamine Production to Alleviate Obesity. J. Food Sci..

[27] Li M., Tang S., Peng X., Sharma G., Yin S., Hao Z., Li J., Shen J., Dai C. (2024). Lycopene as a Therapeutic Agent against Aflatoxin B1-Related Toxicity: Mechanistic Insights and Future Directions. Antioxidants.

[28] Meneguelli T.S., Mishima M.D.V., Hermsdorff H.H.M., Martino H.S.D., Bressan J., Tako E. (2024). Effect of Carotenoids on Gut Health and Inflammatory Status: A Systematic Review of in Vivo Animal Studies. Crit. Rev. Food Sci. Nutr..

[29] Sahin K., Orhan C., Yazlak H., Tuzcu M., Sahin N. (2014). Lycopene Improves Activation of Antioxidant System and Nrf2/HO-1 Pathway of Muscle in Rainbow Trout (*Oncorhynchus mykiss*) with Different Stocking Densities. Aquaculture.

[30] Tahir R., Samra, Afzal F., Ghaffar A., Liang J., Shrestha A., Habiba U., Yang S. (2024). Effectiveness of Lycopene Dietary Enrichment for Growth Performance, Digestive Enzymes, Blood Health, Immunity, and Antioxidant Status of Common Carp (*Cyprinus carpio*) against Chronic Glyphosate Toxicity. Aquaculture.

[31] Hussein M.M.A., Elsadaawy H.A., El-Murr A., Ahmed M.M., Bedawy A.M., Tukur H.A., Swelum A.A.-A., Saadeldin I.M. (2019). Endosulfan Toxicity in Nile Tilapia (*Oreochromis niloticus*) and the Use of Lycopene as an Ameliorative Agent. Comp. Biochem. Physiol. Part C Toxicol. Pharmacol..

[32] Juan C.A., Perez de la Lastra J.M., Plou F.J., Perez-Lebena E. (2021). The Chemistry of Reactive Oxygen Species (ROS) Revisited: Outlining Their Role in Biological Macromolecules (DNA, Lipids and Proteins) and Induced Pathologies. Int. J. Mol. Sci..

[33] Sies H., Jones D.P. (2020). Reactive Oxygen Species (ROS) as Pleiotropic Physiological Signalling Agents. Nat. Rev. Mol. Cell Biol..

[34] Nandi A., Yan L.-J., Jana C.K., Das N. (2019). Role of Catalase in Oxidative Stress- and Age-Associated Degenerative Diseases. Oxidative Med. Cell. Longev..

[35] Jomova K., Alomar S.Y., Alwasel S.H., Nepovimova E., Kuca K., Valko M. (2024). Several Lines of Antioxidant Defense against Oxidative Stress: Antioxidant Enzymes, Nanomaterials with Multiple Enzyme-Mimicking Activities, and Low-Molecular-Weight Antioxidants. Arch. Toxicol..

[36] Wang S., Wu H., Zhu Y., Cui H., Yang J., Lu M., Cheng H., Gu L., Xu T., Xu L. (2022). Effect of Lycopene on the Growth Performance, Antioxidant Enzyme Activity, and Expression of Gene in the Keap1-Nrf2 Signaling Pathway of Arbor Acres Broilers. Front. Vet. Sci..

[37] Jiang H., Wang Z., Ma Y., Qu Y., Lu X., Luo H. (2015). Effects of Dietary Lycopene Supplementation on Plasma Lipid Profile, Lipid Peroxidation and Antioxidant Defense System in Feedlot Bamei Lamb. Asian Australas. J. Anim. Sci..

[38] Ismail R.F., Hamed M., Sayed A.E.-D.H. (2023). Lycopene Supplementation: Effects on Oxidative Stress, Sex Hormones, Gonads and Thyroid Tissue in Tilapia *Oreochromis niloticus* during Harness^®^ Exposure. Front. Physiol..

[39] Sahin K., Yazlak H., Orhan C., Tuzcu M., Akdemir F., Sahin N. (2014). The Effect of Lycopene on Antioxidant Status in Rainbow Trout (*Oncorhynchus mykiss*) Reared under High Stocking Density. Aquaculture.

[40] Xuan Y., Wu D., Zhang Q., Yu Z., Yu J., Zhou D. (2024). Elevated ALT/AST Ratio as a Marker for NAFLD Risk and Severity: Insights from a Cross-Sectional Analysis in the United States. Front. Endocrinol..

[41] Chinnappan R., Mir T.A., Alsalameh S., Makhzoum T., Alzhrani A., Al-Kattan K., Yaqinuddin A. (2023). Low-Cost Point-of-Care Monitoring of ALT and AST Is Promising for Faster Decision Making and Diagnosis of Acute Liver Injury. Diagnostics.

[42] Chinnappan R., Mir T.A., Alsalameh S., Makhzoum T., Adeeb S., Al-Kattan K., Yaqinuddin A. (2023). Aptasensors Are Conjectured as Promising ALT and AST Diagnostic Tools for the Early Diagnosis of Acute Liver Injury. Life.

[43] Almroth B.C., Johnsson J.I., Devlin R., Sturve J. (2012). Oxidative Stress in Growth Hormone Transgenic Coho Salmon with Compressed Lifespan—A Model for Addressing Aging. Free Radic. Res..

[44] Zheng X., Bai Z., Wang T., Romeiro F.G., Mancuso A., Philips C.A., Wong Y.J., Nery F.G., Qi X. (2023). Human Albumin Infusion for the Management of Liver Cirrhosis and Its Complications: An Overview of Major Findings from Meta-Analyses. Adv. Ther..

[45] Lam A.H., King J.D. (2024). Toxin-Induced Liver Injury and Extracorporeal Treatment of Liver Failure. Adv. Kidney Dis. Health.

[46] Yancheva V., Velcheva I., Stoyanova S., Georgieva E. (2016). Histological Biomarkers in Fish as a Tool in Ecological Risk Assessment and Monitoring Programs: A Review. Appl. Ecol. Environ. Res..

[47] Hao M., Liu R. (2019). Molecular Mechanism of CAT and SOD Activity Change under MPA-CdTe Quantum Dots Induced Oxidative Stress in the Mouse Primary Hepatocytes. Spectrochim. Acta Part A Mol. Biomol. Spectrosc..

[48] Ma Q. (2013). Role of Nrf2 in Oxidative Stress and Toxicity. Annu. Rev. Pharmacol. Toxicol..

[49] Zou J., Secombes C. (2016). The Function of Fish Cytokines. Biology.

[50] Tanaka T., Narazaki M., Kishimoto T. (2014). IL-6 in Inflammation, Immunity, and Disease. Cold Spring Harb. Perspect. Biol..

[51] Jang D., Lee A.-H., Shin H.-Y., Song H.-R., Park J.-H., Kang T.-B., Lee S.-R., Yang S.-H. (2021). The Role of Tumor Necrosis Factor Alpha (TNF-α) in Autoimmune Disease and Current TNF-α Inhibitors in Therapeutics. Int. J. Mol. Sci..

[52] Sharifinejad N., Zaki-Dizaji M., Sepahvandi R., Fayyaz F., Dos Santos Vilela M.M., ElGhazali G., Abolhassani H., Ochs H.D., Azizi G. (2022). The Clinical, Molecular, and Therapeutic Features of Patients with IL10/IL10R Deficiency: A Systematic Review. Clin. Exp. Immunol..

[53] Sanjabi S., Oh S.A., Li M.O. (2017). Regulation of the Immune Response by TGF-β: From Conception to Autoimmunity and Infection. Cold Spring Harb. Perspect. Biol..

[54] Di Mascio P., Kaiser S., Sies H. (1989). Lycopene as the Most Efficient Biological Carotenoid Singlet Oxygen Quencher. Arch. Biochem. Biophys..

[55] Kulawik A., Cielecka-Piontek J., Zalewski P. (2023). The Importance of Antioxidant Activity for the Health-Promoting Effect of Lycopene. Nutrients.

[56] Zhou M., Liu H., Lu B., Li B., Huang W., Song H., Cai W., Tan B., Yang Y., Dong X. (2024). Effect of Dietary Lycopene Supplementation on Growth, Antioxidant and Immunity of Juvenile Hybrid Grouper (♀ *Epinephelus fuscoguttatus* × ♂ *E. lanceolatus*) Fed with High Lipid Diets. Aquac. Rep..

[57] Zhi X., Gu Y., Bao Y., Zhao W., Liu X., Zhu J., Cheng H., Mao D., Zhu T., Sun P. (2025). Lycopene Alleviates High Carbohydrate Diet Induced Hepatic Steatosis in Black Seabream (*Acanthopagrus schlegelii*): Potential for Nutritional Intervention. Aquac. Rep..

[58] Heun F., Niebuhr T., Gutierrez Bautista A., Wiedmann F., Verhaar N., Kästner S., Feige K., Schmidt C. (2023). Treatment of a Paroxysmal Atrioventricular Block by Implantation of a Bipolar, Single-Chamber Cardiac Pacemaker in a Donkey. Animals.

[59] Grygiel-Górniak B. (2014). Peroxisome Proliferator-Activated Receptors and Their Ligands: Nutritional and Clinical Implications—A Review. Nutr. J..

[60] Wang J., Geng T., Zou Q., Yang N., Zhao W., Li Y., Tan X., Yuan T., Liu X., Liu Z. (2020). Lycopene Prevents Lipid Accumulation in Hepatocytes by Stimulating PPARα and Improving Mitochondrial Function. J. Funct. Foods.

